# Cosmeceuticals: A Review of Clinical Studies Claiming to Contain Specific, Well-Characterized Strains of Probiotics or Postbiotics

**DOI:** 10.3390/nu16152526

**Published:** 2024-08-02

**Authors:** Ioannis M. Theodorou, Dorothea Kapoukranidou, Markos Theodorou, Joulia K. Tsetis, Alexandra Eleftheria Menni, Georgios Tzikos, Stella Bareka, Anne Shrewsbury, George Stavrou, Katerina Kotzampassi

**Affiliations:** 1 Hellenic Institute for the Study of Sepsis (HISS), 11528 Athens, Greece; itheodorou@med.uoa.gr; 2 Department of Physiology, Faculty of Medicine, Aristotle University of Thessaloniki, 54636 Thessaloniki, Greece; dkapoukr@gmail.com; 3 Uni-Pharma S.A., 14564 Athens, Greece; theodor@uni-pharma.gr (M.T.); jtsetis@uni-pharma.gr (J.K.T.); 4 Department of Surgery, Aristotle University of Thessaloniki, 54636 Thessaloniki, Greece; alexmenn@auth.gr (A.E.M.); gtziko@auth.gr (G.T.); stella.mpareka@gmail.com (S.B.); a_shrewsbury@yahoo.com (A.S.); 5 Department of Surgery, Addenbrooke’s Hospital, Cambridge CB2 2QQ, UK; stavgd@gmail.com

**Keywords:** cosmeceuticals, dermocosmetics, probiotics, postbiotics, skin health, topical use

## Abstract

The skin serves as a critical barrier against external threats—dehydration, ultraviolet exposure, and infections—playing a significant role in internal homeostasis and moisture retention. Additionally, and equally importantly, it interacts dynamically with the complex microbiome resident in it, which is essential for maintaining skin health. Recent interest has focused on the use of probiotics and postbiotics, besides their ability to modulate the skin microbiome, to enhance barrier function, and exhibit anti-inflammatory properties, to be involved in skincare, by having the potential to improve skin hydration, elasticity, and overall appearance, as well as in reducing signs of aging, such as wrinkles and fine lines. The products—being a combination of a cosmetic regime plus probiotic[s] or postbiotic[s]—are named cosmeceuticals. However, to comply with the regulations for the characterization of a microorganism as a specific probiotic strain, the pro- or postbiotics incorporated into the cosmetic regime should be both genetically and phenotypically defined. Thus, in this review, we present 14 published clinical trials using such cosmetic products with specific, well-characterized strains of probiotics or postbiotics applied to volunteers with healthy skin. Looking at the results of these studies collectively, we can say that these genetically and phenotypically defined strains of either live or inanimate bacteria and/or their components seem to keep the treated skin at least fully hydrated, with intact epithelial tone, increased radiance, and with decreased wrinkle depth, while normalizing the commensal skin microbiota. Future advancements in personalized skin care may lead to genomic sequencing and metabolomics to tailor probiotic and postbiotic treatments to individual skin microbiomes, promising a new frontier in cosmeceuticals.

## 1. Introduction

The skin, the body’s largest and most complex organ, serves as the outermost protective barrier against external threats by playing a crucial role in internal homeostasis by means of exerting sensation, regulation, and protection from dehydration, external aggressions—such as ultraviolet exposure and temperature changes—as well as infections [1,2,3]. Additionally, the skin is a dynamic organ that closely interacts with a complex ecosystem of microorganisms, known as the skin microbiome. It houses, in a symbiotic relationship, up to one billion microbiota per square centimeter, including bacteria, fungi, viruses, and mites, which are absolutely essential for maintaining healthy skin by means of protecting against pathogenic microbes, modulating immune responses, and maintaining skin barrier function [4,5,6,7].

The diversity and abundance of these microbial taxa vary considerably between individuals, due to genetic factors, age, gender, diet, lifestyle, and between the different sites on an individual’s skin surface, the latter being directly related to the skin type and location. Thus, we recognize moist, dry, and sebaceous or oily skin areas. Moist areas are mainly colonized by *Staphylococcus* and *Corynebacteria* species, as well as the fungus *Malassezia*, while the axilla is also colonized by Gram-positive bacteria of the genera *Micrococcus* and *Cutibacterium acnes* (formerly known as *Propionibacterium acnes*). Dry areas additionally host *Corynebacterium*, *Enhydrobacter*, and *Streptococcus*, while oily areas, house mainly the lipophilic *Cutibacterium* species, as well as *Staphylococcus* and *Malassezia* species [8,9,10].

Besides its contribution to restoring a balanced environment, the healthy microbiome produces metabolites that are crucial in maintaining skin moisture, elasticity, and resilience, such as organic acids, polysaccharides, and different proteins—mainly enzymes involved in the skin metabolic processes, such as hyaluronic acid production, ceramide synthesis through sphingomyelinase and the stimulation of melanocytes [4]. *Staphylococcus epidermidis*, a commensal bacterium, is probably the most typical example of this process, exhibiting interesting interactions with glycerol, which is a significant component of the skin’s outermost layer of the epidermis, the stratum corneum. It metabolizes glycerol to by-products such as lactic acid, which can assist in maintaining an acidic skin environment, inhibiting the growth of pathogenic bacteria. Glycerol attracts water into the stratum corneum, thus enhancing its hydration and consecutively the preservation of the lipid matrix integrity in the stratum corneum, essential for avoiding trans-epidermal water loss, finally making the skin more flexible and less prone to cracking. Additionally, and more useful teleologically, by preserving skin hydration, it creates an environment that supports the multiplication of beneficial bacteria like *S. epidermidis*, which produce antimicrobial peptides that in turn inhibit the colonization of pathogenic bacteria such as *Staphylococcus aureus* [2,11,12,13]. Another ability of *S. epidermidis* is the stimulation of the production of ceramides in the skin, by producing the enzyme sphingomyelinase, which hydrolyzes the sphingomyelin present in the skin cell membranes, thus releasing ceramides, the outmost component of the lipid matrix of stratum corneum and plays a crucial role in maintaining the skin’s osmotic barrier, thus improving hydration, and avoiding trans-epidermal water loss [14,15,16,17].

These few examples are informative of the inherent ability of commensal bacteria to keep the skin healthy. However, the skin is not in a “closed” laboratory, untouched by genetic and external factors. It not only reflects the general health status but mirrors physical aging, too—the two main types of which are natural aging, and photoaging. Natural or physical aging is in part genetically programmed, however, other internal components of the body, such as hormonal and immunological status, as well as psychological stress are strong additional endogenous factors that can also affect skin quality [1,18,19,20]. Beyond these, environmental factors such as smoking, lifestyle, changes in skin microbiota of whatever etiology, mechanical damage to tissue, and various other factors result in the already reduced number of fibroblasts producing fewer extracellular matrix components. The most significant of these is type I collagen, the reduced amount of which results in a thinner dermal matrix, reduced elasticity, and increased dehydration—most notably manifested by wrinkles and skin relaxation, laxity, and a rough-textured appearance [21,22,23,24,25,26]. At the same time, and supplementary to physical aging, the extent and frequency of ultraviolet radiation exposure speed up skin aging by means of the aberrant reactive oxygen species generation which leads to changes in the microbiome, raised skin pH, decreased collagen levels, and, mainly, an altered immune response [12,27].

Numerous experimental and ex vivo studies have shown that efforts to manipulate the skin bacterial inhabitants, replacing them with healthy ones, namely probiotics [28,29]—given either orally or topically—result in a degree of microbiome restoration, thus reducing inflammation, while enhancing immunomodulation and the epithelial barrier function through the upregulation of tight junction genes [6,30]. The same is also true for the use of non-viable bacteria, cell wall fragments, or their metabolites, now collectively named postbiotics [6,31,32,33,34,35,36,37].

Enhancement of barrier function, achieving a reduction in trans-epithelial water loss, increase in immunomodulatory properties, wound healing, and possible stimulation of collagen and ceramides production—otherwise recognized as “exerting skin-rejuvenating properties”—are each a good reason for applying probiotics and/or postbiotics to the skin and has attracted considerable attention from both scientific communities and dermo-cosmetic industry.

The US Federal Food, Drug, and Cosmetic Act defines cosmetics as “*articles intended to be… applied to the human body...for beautifying, promoting attractiveness, or altering the appearance*” [FD&C Act, sec. 201(i)], which definition was further clarified by the Modernization of Cosmetics Regulation Act of 2022 (MoCRA) and amended to include “*a preparation of cosmetic ingredients with a qualitatively and quantitatively set composition for use in a finished product*” [FD&C Act, sec. 361]. On the other hand, European Regulation No 1223/2009 defines cosmetics as “any substance or mixture intended to be placed in contact with the external parts of the human body… with a view exclusively or mainly to cleaning, perfuming, changing their appearance, protecting them, keeping them in good condition or correcting body odors”. The same Regulation [improving the 1976 Directive 76/768/EC] strengthens the safety of cosmetic products by the introduction of the notion of “responsible person”, having the obligation of reporting serious undesirable effects. Additionally [EU No 655/2013], underlines that the safety of a cosmetic product must be demonstrated and data on microbiological quality must be included in the Cosmetic Product Safety Report.

Although there are numerous products now marketed under the label “containing probiotics” or “containing probiotic lysate” there is reason for considerable caution. To comply with regulations for the characterization of a bacterium as a specific probiotic strain, both genetically and phenotypically, documented and peer-reviewed publications of experiments defining the rationale of their intended use are required [27,38,39]. Furthermore, a postbiotic must be derived from a well-defined microorganism or combination of microorganisms for which genomic sequences are known and prepared using a delineated technological process of biomass production and inactivation, which can be reliably reproduced [3,37,40]. The aim of this review is to select from a high volume of publications only the clinical studies fulfilling the above prerequisites—that is the microorganism used must be well characterized as a specific probiotic strain, live or inanimate [postbiotic], genetically and phenotypically identified, and its specific properties to have been documented experimentally first, the results having been published in peer-reviewed publications.

## 2. Searching the Literature and Findings

A detailed literature search was performed in PubMed; search terms included: topical use or application, cosmetics, cosmeceuticals, probiotics, postbiotics, skin or dermal, randomized clinical trials, clinical studies, and reviews. Preference was given to clinical trials; but all prior meta-analyses, reviews, or narrated reviews were used as possible data sources [Supplementary Materials, Flow Chart].

The trials finally used were only those that referred to cosmetic products [creams, shampoos, lotions] containing probiotics or postbiotics of a clearly identifiable strain [either single or multiple strains], which was applied to healthy-skin volunteers—all skin diseases needing pharmaceutics for systemic treatment were excluded. The final results of the trials—objected quantitative measurements—were compared with those of day 0 [baseline] for the same individual or the corresponding subjects of a placebo-treated group, as each clinical study had been designed by the researchers.

A total of 14 papers were found to fulfill the above criteria; 5 papers referred to the use of cosmetics incorporating probiotics and 9 papers referred to cosmetics incorporating postbiotics.

## 3. Cosmetics with Probiotics [Table 1]

### 3.1. Lactiplantibacillus plantarum HY7714

An early study from the Yakult Co., Yongin, Republic of Korea revealed that the orally taken probiotic formula with *L. plantarum* HY7714 exerts excellent protective effects on the skin of ultraviolet B-irradiated hairless mice against photoaging and dryness, thus achieving for the first time the characterization of a nutricosmetic formula [41,42].

In a double-blind, randomized, clinical study on 110 women aged between 41 and 59 years with Corneometer-confirmed dry skin and wrinkles [readings below 48 arbitrary units and crow’s feet greater than grade 4], the *L. plantarum* HY7714 [10^10^ cfu/d] or placebo was given for 12 wks, the parameters of skin hydration, elasticity, gloss, and wrinkles being tested once per four weeks. The probiotic formulation isolated from the women’s breast milk was found to exert multiple aesthetically beneficial effects: improved facial skin gloss and decreased wrinkle depth [assessed through a 3D skin imaging system] in week 12, with an increased elasticity of 13.2% in week 4 and 21.7% at the end of treatment. Trans-epidermal water loss was found significantly decreased, in parallel with an increase in the facial [*p* < 0.01] and forearm skin moisture content [*p* < 0.05] by week 12, all findings in relation to placebo. These results came following earlier extended experimentation by the authors’ group on hairless mice, in which, among other things, they found that skin moisture content increased in association with ceramide levels—through the regulation of serine palmitoyl transferase and ceramidase expression in the skin—and that the procollagen expression also increased through a matrix metalloproteinase ((MMP)-1) [35].

### 3.2. Nitrosomonas eutropha (D23)

Lee NY et al. highlighted the anti-proliferative role of a purified strain of *N. eutropha* (D23)—an ammonia-oxidizing bacteria-generating nitrite and nitric oxide—on keratinocytes, reducing the hyperproliferative keratinization as well as the overall cutaneous inflammatory state and improving the skin xerosis-related pathologies, such as atopic dermatitis and keratosis pilaris [43]. Although two other clinical trials have further been conducted on skin diseases no publications exist, yet.

Notay et al. [44], based on the known beneficial effects of nitric oxide, such as reduced inflammation, increased vasodilation, and pathogenic bacteria killing, investigated the perspective of restoring aging parameters, that is facial wrinkle depth and severity, skin radiance, and pigmentation, after topical application of live *N. eutropha*, in aerosolized form.

Twenty-nine volunteers, after a washout week, received *N. eutropha* in buffer, twice daily, at a concentration of 1 × 10^9^ cells/mL (the first 10 participants), and at a concentration of 8 × 10^9^ cells/mL (the remaining participants). High-resolution facial photographs were obtained before and at treatment termination, only a week later. Facial wrinkles, quantified by means of algorithm-based modeling revealed a significant improvement in those who received the high-concentration product; radiance improved with both low and high concentrations, while there was no overall change in intensity of pigmentation. The authors reported a further schedule to evaluate possible facial microbiome changes, but the results have not yet been published.

### 3.3. Lactiplantibacillus pentosus KCA1

Onwuliri V, et al. [45] from Nnamdi Azikiwe University, Awka, Nigeria, having previously analyzed the most predominant genera of the human axilla in Nigerians [46], investigated the topical application of a cream containing the *L. pentosus* KCA1 to decrease the malodor volatile substances producing bacteria; the *Corynebacterium striatum*, *C. jeikeium* and *Staphylococcus haemolyticus*, *Staphylococcus hominis*, and *S. lugdunensis*, considered to be the most implicated.

Lyophilized *L. pentosus* KCA1, a probiotic of Nigerian origin [47], in a weight of 12 g was gently mixed with 75 g of an oil-based balm of coconut and lavender oil, plus cocoa and shea butter; this 87 g formula was used by each of the 25 young adult volunteers—13 females and 12 males—applied to the armpit twice daily for 14 days; skin swabs were collected on days 0 and 14. Although monitoring of physical activities as well as the use of antiseptic soap and deodorants and showers taken is impossible as it was based only on self-reporting, all participants reported an absence of axillary malodor during the treatment period.

The bacterial metabolic functional genes encoding pyridoxal phosphate-dependent enzymes revealed a significant down-regulation in the biotransformation of the odor precursor, after application of the *L. pentosus* KCA1 cream. Additionally, the application of this cream led to a significant decrease in the odor-producing *Corynebacterium* species, in both genders; *Corynebacteria* spp., as striatum and jeikeium were found significantly decreased by 96% and 73%, respectively, as also *S. hominis* by 20.8% in males. It is of interest that *C. acnes* also decreased by 46% in males and by over 77% in females, while *Lactobacillus* species generally increased in both diversity and abundance by over 385%.

### 3.4. Lactiplantibacillus plantarum LB244R

An oily texture, semi-solid formulation used as a tracer for the topical application of live *L. plantarum* LB244R^®^ bacteria, at a concentration of 1 × 10^9^ CFU/g [48]. *L. plantarum* LB244R^®^ was selected due to its known antibacterial activity toward S. aureus clonal type 1 and its capacity to improve both atopic skin as well as aging skin [49], while the oily tracer included shea butter, refined oils of rapeseed, jojoba, and sunflower seed, hydrogenated olive oil, sunflower hybrid oil, and tocopherol. The probiotic regime was given a 24 mo shelf-life when kept refrigerated, and no preservatives were needed, due to the oily tracer.

This probiotic-containing ointment was tested on 21 postmenopausal women, aged 49 to 62 years with “normal” to dry skin [Fitzpatrick skin phototypes II to IV]. These women were instructed to apply the formula and massage until total absorption, on a cleaned facial skin, for 56 consecutive days, twice daily. The women were assessed by an experienced dermatologist on days 0, 28, and 56, to confirm the anti-aging potential of the probiotic ointment. Clinical evaluation, skin ultrasonography, and skin biomechanical property assessment, in relation to baseline, revealed an improvement in crow’s feet, spot score, smoothness score, and complexion radiance, an increase in skin hydration, firmness and elasticity as well as dermal density and a decrease in the trans-epidermal water loss and the thickness of the sub-epidermal low echogenic band. However, there was no placebo-treatment group, this being a serious limitation of the study, since the other ingredients of the ointment, such as shea butter, seed oil, jojoba oil, olive oil, sunflower oil, and tocopherol are also known to exert hydration and anti-aging properties [48].

### 3.5. Lactiplantibacillus plantarum LB244R

A few months later, the same authors who had tested *L. plantarum* LB244R in an oily tracer including shea butter, refined oils of rapeseed, jojoba, and sunflower seed, hydrogenated olive oil, sunflower hybrid oil, and tocopherol with positive results, but without placebo group [48] performed a new study with a control group at that time [50].

In this newer study, they investigated the commercially available ointment containing 1 × 10^9^ CFU/g live *L. plantarum* LB244R, isolated from fermented cabbage, in an oily tracer of Byturospermum parkii butter, Simmondsia chinensis seed oil, Brassica campestris seed oil, hydrogenated vegetable oil, Helianthus annuus hybrid oil, Prunus amygdalus dulcis oil, tocopherol, and Helianthus annuus seed oil or the vehicle ointment without bacteria as placebo. Forty-six postmenopausal females, having a mean age of 58.7 years, were randomly assigned to apply either the test ointment or placebo on previously cleaned skin of the face and periocular area, twice daily for 56 consecutive days. They were instructed to massage the product until complete absorption and come for assessment mid-period and at the end of the study.

The probiotic-containing ointment was found to bring significant improvement in relation to placebo to the subepidermal low echogenic band thickness and dermal density, assessed by ultrasonography; the skin firmness and elasticity, assessed by a dual-cutometer apparatus; the trans-epidermal water loss, skin hydration, and pH; the collagen fiber perimeter and density, assessed by confocal microscopy. Additionally, from the clinical evaluation, the crow’s feet wrinkles, the spots, the skin smoothness, and the complexion radiance scores were found significantly improved in comparison to baseline. However, when comparing the final effects [day 56] of the test ointment versus placebo, the probiotic ointment was found to work significantly better in improving the subepidermal low echogenic band thickness, the dermal density, the skin hydration, and the crow’s feet wrinkle score, only [50].

**Table 1:** Cosmetics with probiotics.

Live Bacteria	Applied on	Duration	Placebo Group	Findings	First Author [Reference]
*L. plantarum* HY7714 10^10^ cfu/d	Face	12 wks	Yes	Increased facial skin gloss and skin moisture content Decreased wrinkle depth Decreased TEWL	Lee DE [35]
*N. eutropha* (D23) 8 × 10^9^ cells/mL	Face	1 wk	No	Increased facial radiance Decreased facial wrinkles Decreased the intensity of pigmentation	Notay M [44]
*L. pentosus* KCA1 lyophilized,12 g	Armpit	14 d	No	Absence of axillary malodor during the treatment Decrease in odor-producing Corynebacterium species	Onwuliri V [45]
*L. plantarum* LB244R 1 × 10^9^ cfu/g	Face	56 d	No	Increased skin hydration, firmness, elasticity and density Increased smoothness score and complexion radiance Decreased in crow’s feet, spot score and TEWL	Falholt-Elvebakken H [48]
*L. plantarum* LB244R 1 × 10^9^ cfu/g	Face	56 d	Yes	Increased skin firmness, density, elasticity and smoothness Increased skin hydration, pH and complexion radiance score Decreased crow’s feet wrinkles and TEWL	Elvebakken HF [50]

TEWL: trans-epithelial water loss.

## 4. Cosmetics with Postbiotics [Table 2]

### 4.1. Streptococcus thermophilus S244, Dead by Sonication

As early as 1999, Di Marzio et al. [51] revealed that bacteria of *S. thermophilus*, dead by sonication, when added to a keratinocytes culture were able to induce a significant increase in ceramide levels, in a time-dependent manner. The same result was confirmed after topical application of sonicated bacteria on the forearm skin of 17 healthy subjects for a 7-day period. These findings of increased ceramide levels in the stratum corneum, the outmost layer of the human epidermis, and thus of the clinical effect of increased hydration, were attributed to sphingomyelin hydrolysis, by means of the high levels of bacterial neutral sphingomyelinase from *S. thermophilus* S244; in turn, the increased ceramide levels could result in the enhancement of lipid barrier function and thus to stratum corneum flexibility, slowing down the skin aging process. For further documentation of the ability of *S. thermophilus* to induce a very significant increase in skin ceramide levels, a cream containing purified sphingomyelinase [Avant Garde, Sigma Tau, Pomezia, Rome, Italy] from *Bacillus cereus* [Sigma] was applied as a placebo; the results being comparable.

The same in vivo study was then later repeated with 20 healthy women in order to document improvement in skin hydration [52]. A sonicated *S. thermophilus* S244 bacteria-containing cream [Centro Ricerche YOMO, Italy] was topically applied twice daily to one forearm and the cream only to the other [control], daily for 15 consecutive days during the autumn [to avoid UV radiation exposure], on females over 60 years old, with a healthy skin. Trans-epidermal water loss was found unaffected between treated and controls, meaning that the normal permeability barrier still exists; but forearm skin hydration was found to significantly increase in the treated site in parallel with increased skin ceramides, findings actually related to the presence of S. thermophilus sphingomyelinase, which enabled maintenance of the dermal protective barrier and increase in resistance against age-related skin xerosis.

### 4.2. Bifidobacterium longum Reuter Lysate

Guéniche et al. [53], having already demonstrated, in both in vitro and in ex vivo human skin explant models, the beneficial effects of *B. longum* reuter lysate to improve sensitive skin, tested it as a cream formula containing 10% bacterium lysate, in a randomized double-blind study on the face, arms, and legs of 66 female volunteers, twice a day for two months. They had previously confirmed that the ultrasound-inactivated *B. longum* reuter lysate suspension of 5% in the aqueous medium led to an improvement in parameters relating to inflammation—a decrease in vasodilation, edema, mast cell degranulation, and TNF-alpha release—and more specifically of neurogenic inflammation, since it significantly inhibited capsaicin-induced CGRP release by neurons. The same regime, in a pilot clinical trial, was also found to lead to increased skin resistance to physical and chemical aggression in relation to controls. Given these anti-inflammatory properties, they tested this postbiotic in females with reactive skin, that is skin demonstrating excessive sensitivity to physical conditions like heat, cold, wind, or topically applied chemical products, resulting in a decrease in the skin’s self-repair/rejuvenation ability.

Treated volunteers exhibited a significant improvement: increased skin resistance, that is a decrease in skin sensitivity assessed by stinging test [*p* = 0.002], and an improvement in skin barrier recovery [trans-epidermal water loss less than 15 g/cm^2^/h, *p* = 0.004] assessed by repeated tape-stripping at the beginning, after a month and at the end of the 2nd month. Additionally, volunteers’ self-assessment emphasized a decrease in skin roughness and dryness [*p* = 0.03], after just a month of treatment, versus controls. These new findings, in correlation with those previously reported, led the authors to conclude that the postbiotic *B. longum* reuter regime decreases skin sensitivity by reducing neuron reactivity.

### 4.3. Lactobacillus brevis DSM17250

The L. brevis DSM17250 was selected among several hundred Lactobacillus strains banked in the DSMZ [German Collection of Microorganisms and Cell Cultures (Braunschweig, Germany) with the criterion of being able to stimulate *S. epidermidis* growth in vitro. This specific bacterium, *L. brevis* DSM17250, was further found to exhibit an anti-inflammatory effect on human keratinocytes in vitro, as assessed by the secretion of the pro-inflammatory cytokine IL-1α, while acting specifically on commensal skin *S. epidermidis* DSM20044, enhancing the growth both in vitro and in vivo [54].

It was then used in a human study. Thirty volunteers, mainly females, with a mean age of 39 years, with skin xerosis at their lower legs [tibia], were allocated either to a standard hydrophilic ointment containing 0.88 mg/g of the *L. brevis* DSM17250 extract or to hydrophilic ointment alone, twice a day for a month. Treatment or the placebo ointment was applied to the skin corresponding to the right anterior tibia area, while the same skin area of the left side was left totally untreated, to serve as an internal control. All participants daily self-assessed regarding dryness, roughness, a feeling of tightness, itching, and burning in relation to the contralateral leg; and by the dermatologist on day 0 and then on days 7, 14, and 28, with respect to skin barrier function [trans-epidermal water loss], with clinical scores of redness, roughness, scaling and cracks/fissures; finally, an 8 cm^2^ skin tape was thoroughly attached to the skin under study, to collect the bacteria adhering to it, for the assessment of differences over the course of treatment [54].

The postbiotic cream was found by self-evaluation test to significantly improve xerosis parameters—collectively by about 70% in relation to 57% in placebo—being parallel to the measurements of trans-epidermal water loss [8.98 g/m^2^/h in the treatment group versus 15.68 g/m^2^/h in placebo, on day 28]. Regarding skin microbiota, besides *S. epidermidis*, other commensals such as Micrococcus, *S. capitis,* and *S. xylosus* were also found, the total number of commensal skin bacteria being significantly increased, from 141 to 399 cfu in the 8 cm^2^ area of stripping tape used [54].

### 4.4. Lactiplantibacillus plantarum-GMNL6—Heat-Killed

Tsai et al. [55] investigated whether heat-killed *L. plantarum*-GMNL6 interacts with skin microbiota resulting in improvement in skin health. Fifteen healthy volunteers applied a “base” cream containing heat-killed L. plantarum-GMNL6 [1 × 10^9^ cells/g cream] to the right cheek and to the left, the cream only, twice a day for two months, after being subjected to skin quality, moisturizing, and color tests.

Skin hydration, assessed by either a Corneometer^®^ CM 825 or a Skicon 200 EX^®^, was found to be significantly better than in the controls, as also occurred with facial skin quality, wrinkles, skin texture, tone, and UV spots, assessed by using Visia (Visia Complexion Analysis, Canfield, Parsippany, NJ, USA); skin erythema and melanin indexes, respectively, assessed by a Derma-Spectrometer were significantly reduced. Finally, the 16S rDNA sequencing used to analyze the bacterial abundance and the bacterial composition in the skin revealed that in the L. plantarum-treated right cheeks Cutibacterium was found to be significantly reduced, while Streptococcus and Staphylococcus showed a higher abundance in relation to the non-treated left cheeks [55].

### 4.5. Lacticaseibacillus paracasei GMNL-653—Heat-Killed

Two years later, they investigated the beneficial role of heat-killed *L. paracasei* GMNL-653 on human scalp health [56]. Twenty-two volunteers used a control shampoo for one month and for the next four months the same shampoo containing the *L. paracasei* GMNL-653 postbiotic. The scalp conditions, i.e., sebum secretion, dandruff formation, and hair growth were evaluated in 15-day intervals, while the scalp skin commensal Malassezia yeasts [*M. restricta* and *M. globose*]—etiologically related to pityriasis and seborrheic dermatitis—as well as the microbiota *C. acnes*, and *S. epidermidis*—known to reciprocally inhibit scalp bacteria—were detected by means of quantitative polymerase chain reaction [PCR], before and after application of control and postbiotic-containing shampoos.

Results showed that oil count and dandruff were significantly decreased, and hair volume statistically increased after treatment, but only in the subgroup of individuals with high levels and not in those with normal values at the beginning. In respect to microbiota, *C. acnes* was found statistically decreased and *M. globosa* increased after treatment in relation to the placebo, given in the first month. There was no correlation between *L. paracasei* and *S. epidermidis* or *M. restricta* in general. However, the presence of *L. paracasei* was positively correlated with *S. epidermidis* only, in the high dandruff subgroup, although *M. restricta* statistically decreased in this subgroup of high dandruff after the use of treatment shampoo.

It is clear that the beneficial effects on sebum secretion and dandruff achieved by shampoo containing the postbiotic *L. paracasei* GMNL-653 seem more significant for those with weak scalp conditions in relation to those with healthy scalps; the valuable role being attributable to the observed microbiota shift and more specifically to abundance changes occurring to *C. acnes* and *S. epidermidis* [56].

### 4.6. L. plantarum AN057, L. casei AN177, and S. thermophilus AN157 Metabolites

The probiotic strains *L. plantarum* AN057, *L. casei* AN177, and *S. thermophilus* AN157—first isolated from Balkanian fermented dairy products and then fully characterized by means of a genomic analysis—were subjected to co-fermentation, resulting in a postbiotic combination of metabolites, named CLS02021 [4]. The authors claim that the above strains were carefully selected, based on their potential to produce, through co-fermentation, specific metabolites, such as lipoteichoic acid, hyaluronic acid, lactic acid, and Sphingomyelinase and indeed in considerable quantities.

Fifty healthy adult females, up to 69 years of age, voluntarily participated in a double-blind, intra-individual, randomized split-face testing study [ClinicalTrials.gov: NCT05048121] lasting four weeks. They were instructed to apply the treatment cream CLS02021 and the placebo, twice daily, provided in blinded tubes having the only indications “Left” and “Right”. Skin nourishment and elasticity, wrinkle depth, sebum production, melanin production, as well as skin sensitivity and cleanliness, were measured on days 0, 14, and 28, along with the safety profile.

A statistically significant improvement was reported between postbiotics and placebo groups regarding skin moisture and elasticity [*p* < 0.0001], pore size, and wrinkle depth [*p* < 0.05], while no difference was presented regarding sebum production, melanin, sensitivity, and cleanliness. The significant increase in skin moisture levels and elasticity is likely to be attributable to sphingomyelinase facilitating the production of ceramides, being the key components of skin barrier integrity maintenance [57], as well as to hyaluronic acid along with lactic acid, which, through their capacity to bind large water volumes, decrease the trans-epidermal water loss and rehydrate the skin, thereby filling in fine lines and reducing the appearance of wrinkles [58].

### 4.7. Epidermidibacterium Keratini (EPI-7) Ferment Filtrate

A novel, rod-shape, aerobic, Gram-positive bacterium of the *Actinobacterium phylum*, *E. keratini* (EPI-7), recently isolated from human epidermal keratinocytes from the cheeks of young females [59], was found to exhibit anti-aging properties in human dermal fibroblasts after being subjected to UV photo-aging. This occurs through the modulation of UV activity on matrix metalloproteinases [60], and through one of the EPI-7 ferment filtrates, orotic acid, known to be crucially involved in pyrimidines biosynthesis [61].

The authors [62] investigated the anti-aging efficacy and safety of the postbiotic EPI-7 ferment filtrate, applied topically for 3 weeks, in relation to the skin microbiome diversity. A total of 55 Korean women of different age groups, between 19 and 60+ years of age enrolled in this randomized, split-face clinical study showed highly significant improvements in skin barrier function, elasticity, and dermal density versus control treatment, possibly related to the restoration of commensal microbiota diversity observed.

Indeed, an increase in the abundance of the commensal bacteria of *Staphylococcus*, *Cutibacterium*, *Corynebacterium*, *Lawsonella*, *Streptococcus*, *Clostridium lactobacillus*, and *Prevotella* spp. was prominent, and more specifically, *Cutibacterium* was found significantly increased in parallel with *Clostridium*, and *Prevotella*. The authors consider that the enhancement of the normally distributed diversity of bacteria species without any alteration in the dominance of any specific microorganism in the skin microbiome is linked to the metabolite orotic acid action, however further analysis and clinical tests are required [62].

Additionally, application of the postbiotic EPI-7 resulted in no observable itching, pain, or erythema.

### 4.8. Lactiplantibacillus plantarum HDB Lysate

The postbiotic *L. plantarum* HDB [probiotic lysate], previously examined in human skin cells as to whether it improves the skin barrier [63], was tested twice a day for 2 weeks on the cheeks of 21 randomly assigned healthy females from Korea, voluntarily participating in this investigation—the other cheek served as control [64]. The skin-hydration parameters recorded before and after treatment or placebo application were then correlated with the skin microbiota of the participants, identified using 16s rRNA gene sequencing.

The postbiotic *L. plantarum* HDB, received from the HDB Cell Bank [Hyundai Bioland Co., Ltd., Ansan, Republic of Korea] named Dermabiotics HDB, was added to an essence cosmetic formula consisting of glycerin, butylene glycol, polyethylene glycol, oils, and preservatives, and this formula was also used as the control. The *L. plantarum* treatment was found to significantly enhance the moisture intensity of the keratin layer surface. Conversely, trans-epidermal water loss and skin redness were significantly reduced in relation to controls, as also seen with skin microbiome versus control: after *L. plantarum* HDB treatment, the α-diversity analysis revealed a significantly decreased richness in species, although the composition of the skin microbiota was very diverse at the beginning, probably due to the range of women’s ages [20–50 years old] [65].

After 2 weeks, the genera of the phylum Proteobacteria, known to dominate in the dry areas of the skin, was found to decrease, the level of decrease matching an improvement in skin condition in general; while the abundance of *Staphylococci* was related to the lowering of the hot flush level; however, from the genus *Lawsonella*, known as skin microbiota related to skin moisture content, *Lawsonella clevelandensis* showed a negative correlation with the keratin layer hydration, and Corynebacterium, a member of the same order, showed a positive correlation with the trans-epidermal water loss. The authors thus confirmed that the Dermabiotics HDB formula may be considered a potential skin microbiome-based cosmetic component [64].

### 4.9. Fermented lysates VHProbi^®^ Mix R

A lotion made from fermented lysates of *Lacticaseibacillus rhamnosus* VHProbi^®^ E06 (E06), *Lacticaseibacillus paracasei* VHProbi^®^ E12 (E12), *Lactiplantibacillus plantarum* VHProbi^®^ E15, and *Lactobacillus helveticus* VHProbi^®^ Y21 was applied to investigate the possibility of enhancing the skin barrier [66].

Previous preliminary vitro studies have shown that the fermented cultures of *L. rhamnosus* VHProbi^®^ E06 (E06) and *L. paracasei* VHProbi^®^ E12 (E12) exerted an antioxidant effect, boosted the proliferation of HaCaT cells, and ameliorated the noxious effects of UV radiation, hydrogen peroxide and *S. aureus* ATCC 25,923 in the HaCaT cell models [66], while the fermented lysate from *L. plantarum* VHProbi^®^ E15 (E15) improved the symptoms of acne [67].

Fifty-two adults with sensitive skin, defined as that based on a positive response to the lactic acid stinging test and having a score ≥ 8 in the Burden of Sensitive Skin [BoSS] questionnaire [66], were eligible to apply the lotion to their faces twice daily for a month. The 3% VHProbi^®^ MixR was incorporated within 100 mL of aqua with propylene glycol, caprylic/capric triglyceride, mineral oil, cetearyl alcohol, PEG-100 glyceryl stearate, stearic acid, glyceryl stearate, 1,2-hexanediol, phenoxyethanol, xanthan gum, chlorophenesin, and L-arginine as preservatives, chelating agents, and thickeners in the lotion.

On day 30, a highly significant decrease [*p* < 0.01] in trans-epithelial water loss, skin redness, redness profile, and increased moisturization was found in relation to day 0. The BoSS questionnaire revealed an improvement of 24.3% from the baseline, while on self-assessment 75% of individuals reported a good or excellent drop in skin swelling, oozing, scabs, and rashes [66].

**Table 2:** Cosmetics with postbiotics.

Bacteria Used	Quantity/ Duration	Applied on	Placebo Group	Findings	Microbiome Modulation	First Author [Reference]
*S. thermophilus* S244 sonicated	— 15 d	Forearm	Yes	Increased skin hydration Increased skin ceramides	-	Di Marzio L [52]
*B. longum* *reuter* lysate	10% cream 2 mo	Face Arms Legs	Yes	Increased skin resistance and skin barrier Decreased skin sensitivity Decrease in skin roughness and dryness	-	Guéniche A [53]
*L. brevis* DSM17250 extract	0.88 mg/g ointment 28 d	Tibia	Internal Control	Decreased TEWL	Increase *S. epidermidis*	Holz C [54]
*L. plantarum GMNL6* heat-killed	1 × 10^9^ cells/g cream 2 mo	Face	Internal Control [split face]	Increased skin hydration Improvement of wrinkles, skin texture, tone and UV spots Decreased skin erythema and melanin indexes	Decreased *Cutibacterium* increased *Streptococcus* *Staphylococcus*	Tsai, WH [55]
*L. paracasei* GMNL-653 heat-killed	— Shampoo 4 mo	Scalp	Baseline	Decreased sebum secretion and dandruff Increased hair volume	Decreased *Cutibacterium* *acnes* *Malassezia globosa*	Tsai WH [56]
*L. plantarum AN057*, *L. casei AN177*, *S. thermophilus AN157* co-fermented metabolites CLS02021	— 4 wks	Face	Internal Control [split face]	Increased skin moisture and elasticity Decreased skin pore size and wrinkle depth	-	Catic T [4]
*E. keratini* *(EPI-7)* fermented filtrate	— 3 wks	Face	Internal Control [split face]	Increased skin barrier function Increased elasticity, and dermal density	Increased *Cutibacterium*, *Clostridium*, *Prevotella*	Kim J [62]
*L. plantarum* *HDB* lysate	— 2 wks	Face	Internal Control [split face]	Increased skin moisture Decreased TEWL Decreased skin redness	Decreased *Proteobacteria*, *Staphylococcii*, *Lawsonella clevelandensis* increased *Corynebacterium acnes*	Kim KM [64]
*L. rhamnosus VHProbi^®^E06* *L. paracasei VHProbi^®^ E12* *L. plantarum VHProbi^®^ E15* *L. helveticus VHProbi^®^ Y21* fermented lysates *VHProbi^®^* Mix R	3% in aquatic solution 1 mo	Face	Baseline	Increase moisturization Increased Burden of Sensitive Skin Decreased TEWL Decreases skin redness, redness profile score Decreased skin swelling, oozing, scabs, rashes	-	Cui, H. [66]

## 5. Discussion

Cosmetic products serve to maintain youthful and healthy skin, as well as to address the signs of aging, by improving the skin’s appearance and resilience. The main functions are [i] barrier repair and hydration, achievable through moisturizers and humectants. Moisturizers, such as hyaluronic acid, glycerin, and ceramides—and fatty acids in general—help to maintain or protect and/or repair skin epithelial barriers against environmental damage, to finally prevent dehydration. Humectants, such as urea and alpha hydroxy acids, attract water to the skin, keeping it moisturized [68,69]; [ii] tissue repair, through anti-aging ingredients promoting cell turnover and stimulating collagen production, thus improving the overall skin texture and the facial oval shape, as occur with retinol and various growth factors as well as stem cell technology, encouraging skin regeneration [70,71]; [iii] protection, against harmful UV radiation, and free radicals in general, causing skin damage and leading to premature aging [72]; [iv] brightening, as occurs with vitamin C, which reduces the appearance of dark spots and evens out skin tone; [v] firming, by different peptides, retinoids and plant extracts, which improve skin elasticity and reduce sagging; and [vi] cleansing, by means of gentle cleansers with balanced pH to remove dirt, oil, and impurities and maintain the skin’s natural barrier. Additionally, there are exfoliation, anti-inflammatory and smoothing agents. Finally, there are products for special purposes or targeted products, such as serums with concentrated ingredients for specific skin concerns such as fine lines, hyperpigmentation, and dehydration; and masks, which provide intensive treatment for a short period, offering hydration, brightening, or soothing benefits [38,73,74,75].

However, the skin houses, in a symbiotic relationship, a complex ecosystem, the skin microbiome; the dynamic interaction with which is absolutely essential for many crucial functions for maintaining healthy skin [4,5,6,7]. Thus, our Greek grandmothers’ secret receipt of full-fat sheep yogurt, mixed with a few drops of olive oil or lemon [depending on skin type] applied as a face mask on Saturday night after the weekly bath and before church on Sunday, is now being transformed into science and commerce. The starter for yogurt was initially the stomach contents of a newborn sheep killed for Easter. Almost a century later, we recognize this as a regime containing *S. thermophilus*, *Lactobacillus delbrueckii* subsp. bulgaricus and *L. casei* and, most probably, as a result of contamination, with other lactic acid bacteria, which produce lactic acid, decreasing pH and causing milk protein to coagulate [76,77].

In recent years, research at any complexity level [from cell cultures to genomic sequencing and metabolomics] has indicated that the incorporation of probiotics or their lysates or bacteria killed by heat, sonication, or other methods or merely cell wall fragments [altogether now called postbiotics] into cosmetic formulations can effectively modulate the skin microbiota, which has become a prominent therapeutic option for treating various skin disorders. Thus, topical applications have attracted considerable attention from scientific and industrial communities motivated by the human desire to be attractive, to fight the signs of age, and to tone down physical damage [53,78,79].

Hundreds of products advertised to protect the skin, mainly the face, or rebalance the skin microbiome and/or reported to enhance skin hydration, improve skin elasticity and radiance, and reduce the fine lines or the extent of wrinkles, are the new norm in the dermo-cosmetic field [9,34,35,80]. They are used worldwide in various “cosmeceutical” formulations such as creams, masks, lotions, serums, cleansers, and so on, and as anti-aging products due to their broad antimicrobial, anti-inflammatory, and antioxidant properties.

Thus, the term cosmeceuticals [as the word nutraceuticals used for orally taken probiotic regimes] means cosmetic products incorporate bioactive ingredients purported to have medical benefits. In early 2023, a publication by Yadav et al. [81] reported that 928 patents had been retrieved from the database after searching with the terms “probiotic” and “cosmetic” in 3 years, most applicants coming from the US, the Republic of China, and the Republic of Korea, but, at least for the US, there are no legal requirements to prove that these products live up to their claims. Thus, several cosmetic companies across the world have probiotic or postbiotic bacteria formulations commercially available, although the bacteria’s origin and their clinical documentation are not always clear from the packaging or website.

However, it is well known from scientific research that not all *Lactobacilli* or *Bifidobacteria*, are considered to be probiotics, no that all have exactly the same probiotic properties. It is thus clear that the label “containing probiotics” or “containing probiotic lysate” says nothing at all. The microorganism used must be characterized as a specific probiotic strain, genetically and phenotypically identified, and its specific properties to have been documented experimentally first, the results having been published in peer-reviewed publications [27,37,38,39]. Furthermore, according to Telesetsky A et al. [82] the probiotic regime must have the same quantity of live bacteria used in the clinical trial, as the commercial product, to benefit the designated target site, and when people are the intended receivers, the delivery technique, dosage, and length of use should be determined through human studies [27,38,82].

In the present review, we selected those clinical studies on cosmeceuticals fulfilling all the above prerequisites. We thus found and analyzed only 14 studies: 5 incorporating probiotics, and 9 postbiotics [Table 1 and Table 2]. The probiotics used were the species *L. plantarum* HY7714, *N. eutropha* (D23), and *L. plantarum* LB244R [two studies], applied to facial skin, and one clinical trial applied to the armpit [*L. pentosus* KCA1]. All four studies on facial skin revealed an improvement in skin appearance [wrinkle depth, skin radiance, and elasticity], while *L. plantarum* HY7714 and *L. plantarum* LB244R also significantly improved skin barrier function, decreasing the trans-epidermal water loss and increasing skin hydration. In the case of *L. pentosus* KCA1, applied to the armpit, it was found to decrease the odor-producing Corynebacterium species, thus achieving a significant improvement in axillary malodor during the treatment.

Nine preparations incorporated the postbiotics: Sonicated *S. thermophilus* S244, *Bifidobacterium longum* reuter lysate, heat-killed *L. plantarum*-GMNL6, the co-fermented *L. plantarum* AN057, *L. casei* AN177, and *S. thermophilus* AN157, E. keratini (EPI-7), *L. plantarum* HDB lysate, and a fermented lysate of *L. rhamnosus* VHProbi^®^ E06, *L. paracasei* VHProbi^®^ E12, *L. plantarum* VHProbi^®^ E15, and *L. helveticus* VHProbi^®^ Y21, all applied to facial skin or the forearm; another preparation, the heat-killed *L. paracasei* GMNL-653, used as a hair shampoo, exhibited positive results in decreasing sebum secretion and dandruff in parallel with a decrease in the abundance of *C. acnes* and *M. globose*, while there was an increase in hair volume. Finally, in the case of *L. brevis* DSM17250, applied on the skin of the tibia, the skin moisture was increased due to the strengthening of the skin barrier and the reduction of the trans-epithelial water loss, attributable to the modulation of commensal bacteria, and especially of *S. epidermidis*.

The 11 regimes for facial and forearm skin use were all found to keep the skin hydrated by strengthening the skin barrier, decreasing trans-epidermal water loss, and improving skin quality. No study directly referred to ceramide or collagen increase, while in five studies the skin microbiome remodeling was described: reduction of Proteobacteria and increase in the abundance of *Cutibacterium* and of *C. acnes*.

Considering the results of these studies collectively, we can say that these thoroughly studied probiotics, or the derived postbiotics, seem to keep the treated skin at least fully hydrated, with intact epithelial tone, positively affecting water loss; furthermore, they exert anti-inflammatory properties against various external attack [UV radiation, free radicals] thus delaying skin aging. Furthermore, some strains are involved in ceramide production as well as in boosting collagen synthesis, through fibroblast proliferation. In any case, a fully hydrated face shows improved elasticity, increased radiance, and decreased wrinkle depth. Most importantly, however, all these “genetically and phenotypically well-studied” cosmeceuticals can effectively regulate the skin microbiota towards a healthier one, their specialized action being absolutely species-specific [36,38].

However, these studies have several inherent drawbacks. First of all, those with the highest number of participants have a maximum of 55 subjects per study group—one on probiotics, with 55 subjects per arm, and 3 on postbiotics with 50, 55, and 52 subjects in total, while the remaining 10 have between 15 and 30 participants. The second drawback is that only three studies have a placebo group, two studies with probiotics, and one with postbiotics. Of the remaining 11, 4 with probiotics compare treatment results with baseline [before—after treatment], the treatment lasting 1 to 8 weeks [4 months for the shampoo study]. The seven studies use the corresponding half of the body for control. Finally, there are only four studies that report the ingredients of the formula used as a tracer. An initial study with probiotics reported a formula containing shea butter, seed oil, jojoba oil, olive oil, sunflower oil, and tocopherol plus the probiotic, the comparison being made before and after treatment. In the next study, the same authors used an oily tracer of *Byturospermum parkii* butter, *Simmondsia chinensis* seed oil, *Brassica campestris* seed oil, hydrogenated vegetable oil, *Helianthus annuus* hybrid oil, *Prunus amygdalus* dulcis oil, tocopherol, and *Helianthus annuus* seed oil, with or without the probiotic, the comparison made according to the split-face method. However, it is questionable as to how one can find differences between probiotics and placebo, the tracer having so many oils known for their beneficial effects on the skin. Finally, in two studies with postbiotics a detailed reference of the ingredients was also reported: glycerin, butylene glycol, polyethylene glycol, oils, and preservatives plus postbiotic was tested through the before-after comparison; and propylene glycol, caprylic/capric triglyceride, mineral oil, cetearyl alcohol, PEG-100 glyceryl stearate, stearic acid, glyceryl stearate, 1,2-hexanediol, phenoxyethanol, xanthan gum, chlorophenesin, L-arginine, chelating agents, and thickeners plus postbiotic or not, in a split-face study.

The skin reflects general health status and aging. As aging progresses, the fine lines become deep wrinkles, crow’s feet, hooded eyelid, crepiness of the periorbital area, a double chin, etc., progressively appear. On the other hand, life expectancy is increasing, and since many people today do not have financial problems for survival, it is a very human reaction, not only for females, to look at their faces and bodies in the mirror and to desire to do something to counteract the effects of aging for themselves [73,80]. The use of beauty products, regardless of their origin, ingredients, manufacturers, and cost, is an integral part of daily care for many throughout the world, as well as a kind of psychotherapy [83,84]. The ongoing use of cosmeceuticals gives hope to the possibility of skin rejuvenation, the smoothing of wrinkles, and the freshening and firming of skin. Humans consent to pay for this—the timeless story of Goethe’s Fausto—but consent to pay for reliable products, both in relation to their ingredients and their effectiveness.

## 6. Future Perspectives

The entrance of cosmeceuticals into the fight against dermo-aging is progressing rapidly, though it is still relatively early days. Probiotics and postbiotics are two distinct entities with a common origin, one from the other, both directly linked to a healthy skin microbiome. While the positive effects of specific probiotic strains on skin health are well-documented in cell line cultures and animal models, translating these findings to human studies presents significant challenges that will necessitate concerted and cooperative efforts involving robust clinical trials, on the part of researchers, industry, and regulatory bodies.

Over and above this, since everybody has a specific and individual microbiome, his “microbiota fingerprint”, implicated in his dermal homeostasis, we believe that in the near future a prescription for personalized cosmeceutical products should be possible after a “rapid test” analysis of the skin microbiome. This would indeed be the new trend in dermo-cosmeceuticals.

## Supplementary Materials

The following supporting information can be downloaded at: https://www.mdpi.com/article/10.3390/nu16152526/s1, Flow Chart.

## References

[1] Guéniche A., Philippe D., Bastien P., Blum S., Buyukpamukcu E., Castiel-Higounenc I. (2009). Probiotics for photoprotection. Derm. Endocrinol..

[2] Grice E.A., Segre J.A. (2011). The skin microbiome. Nat. Rev. Microbiol..

[3] da Silva Vale A., de Melo Pereira G.V., de Oliveira A.C., de Carvalho Neto D.P., Herrmann L.W., Karp S.G., Soccol V.T., Soccol C.R. (2023). Production, Formulation, and Application of Postbiotics in the Treatment of Skin Conditions. Fermentation.

[4] Catic T., Pehlivanovic B., Pljakic N., Balicevac A. (2022). The Moisturizing Efficacy of a Proprietary Dermo-Cosmetic Product (CLS02021) Versus Placebo in a 4-week Application Period. Med. Arch..

[5] Li X., Xing L., Lai R., Yuan C., Humbert P. (2021). Literature mapping: Association of microscopic skin microflora and biomarkers with macroscopic skin health. Clin. Exp. Dermatol..

[6] Rawal S., Ali S.A. (2023). Probiotics and postbiotics play a role in maintaining dermal health. Food Funct..

[7] Lolou V., Panayiotidis M.I. (2019). Functional Role of Probiotics and Prebiotics on Skin Health and Disease. Fermentation.

[8] Dréno B., Araviiskaia E., Berardesca E., Gontijo G., Sanchez Viera M., Xiang L.F., Martin R., Bieber T. (2016). Microbiome in healthy skin, update for dermatologists. J. Eur. Acad. Dermatol. Venereol..

[9] Gueniche A., Perin O., Bouslimani A., Landemaine L., Misra N., Cupferman S., Aguilar L., Clavaud C., Chopra T., Khodr A. (2022). Advances in Microbiome-Derived Solutions and Methodologies Are Founding a New Era in Skin Health and Care. Pathogens.

[10] Egert M., Simmering R. (2016). The Microbiota of the Human Skin. Adv. Exp. Med. Biol..

[11] Coppola S., Avagliano C., Sacchi A., Laneri S., Calignano A., Voto L., Luzzetti A., Berni Canani R. (2022). Potential Clinical Applications of the Postbiotic Butyrate in Human Skin Diseases. Molecules.

[12] Keshari S., Balasubramaniam A., Myagmardoloonjin B., Herr D.R., Negari I.P., Huang C.-M. (2019). Butyric Acid from Probiotic *Staphylococcus epidermidis* in the Skin Microbiome Down-Regulates the Ultraviolet-Induced Pro-Inflammatory IL-6 Cytokine via Short-Chain Fatty Acid Receptor. Int. J. Mol. Sci..

[13] Fluhr J.W., Mao-Qiang M., Brown B.E., Wertz P.W., Crumrine D., Sundberg J.P., Feingold K.R., Elias P.M. (2003). Glycerol regulates stratum corneum hydration in sebaceous gland deficient (asebia) mice. J. Investig. Dermatol..

[14] Imokawa G., Abe A., Jin K., Higaki Y., Kawashima M., Hidano A. (1991). Decreased level of ceramides in stratum corneum of atopic dermatitis: An etiologic factor in atopic dry skin?. J. Investig. Dermatol..

[15] Breiden B., Sandhoff K. (2014). The role of sphingolipid metabolism in cutaneous permeability barrier formation. Biochim. Biophys. Acta.

[16] Rabionet M., Gorgas K., Sandhoff R. (2014). Ceramide synthesis in the epidermis. Biochim. Biophys. Acta.

[17] Samadi A., Nasrollahi S.A., Rostami M.N., Rezagholi Z., Abolghasemi F., Firooz A. (2021). Long-term effects of two 24-hour moisturizing products on skin barrier structure and function: A biometric and molecular study. Health Sci. Rep..

[18] Montagna W., Carlisle K. (1979). Structural changes in aging human skin. J. Investig. Dermatol..

[19] Makrantonaki E., Bekou V., Zouboulis C.C. (2012). Genetics and skin aging. Derm.-Endocrinol..

[20] Pageon H., Azouaoui A., Zucchi H., Ricois S., Tran C., Asselineau D. (2019). Potentially beneficial effects of rhamnose on skin ageing: An in vitro and in vivo study. Int. J. Cosmet. Sci..

[21] Margolis D.J., Kruithof E.K., Barnard M., Howe K., Lazarus G.S. (1996). Fibrinolytic abnormalities in two different cutaneous manifestations of venous disease. J. Am. Acad. Dermatol..

[22] Watson R.E., Griffiths C.E., Craven N.M., Shuttleworth C.A., Kielty C.M. (1999). Fibrillin-rich microfibrils are reduced in photoaged skin. Distribution at the dermal-epidermal junction. J. Investig. Dermatol..

[23] Shuster S., Black M.M., McVitie E. (1975). The influence of age and sex on skin thickness, skin collagen and density. Br. J. Dermatol..

[24] Russell-Goldman E., Murphy G.F. (2020). The Pathobiology of Skin Aging: New Insights into an Old Dilemma. Am. J. Pathol..

[25] Zhang S., Duan E. (2018). Fighting against Skin Aging: The Way from Bench to Bedside. Cell Transplant..

[26] Cole M.A., Quan T., Voorhees J.J., Fisher G.J. (2018). Extracellular matrix regulation of fibroblast function: Redefining our perspective on skin aging. J. Cell Commun. Signal..

[27] Dou J., Feng N., Guo F., Chen Z., Liang J., Wang T., Guo X., Xu Z. (2023). Applications of Probiotic Constituents in Cosmetics. Molecules.

[28] Hill C., Guarner F., Reid G., Gibson G.R., Merenstein D.J., Pot B., Morelli L., Canani R.B., Flint H.J., Salminen S. (2014). Expert consensus document: The International Scientific Association for Probiotics and Prebiotics consensus statement on the scope and appropriate use of the term probiotic. Nat. Rev. Gastroenterol. Hepatol..

[29] Callewaert C., Knödlseder N., Karoglan A., Güell M., Paetzold B. (2021). Skin microbiome transplantation and manipulation: Current state of the art. Comput. Struct. Biotechnol. J..

[30] Yu X., Wei M., Yang D., Wu X., Wei H., Xu F. (2023). *Lactiplantibacillus plantarum* Strain FLPL05 Promotes Longevity in Mice by Improving Intestinal Barrier. Probiotics Antimicrob. Proteins.

[31] Salminen S., Collado M.C., Endo A., Hill C., Lebeer S., Quigley E.M.M., Sanders M.E., Shamir R., Swann J.R., Szajewska H. (2021). The International Scientific Association of Probiotics and Prebiotics (ISAPP) consensus statement on the definition and scope of postbiotics. Nat. Rev. Gastroenterol. Hepatol..

[32] Vinderola G., Druart C., Gosálbez L., Salminen S., Vinot N., Lebeer S. (2023). Postbiotics in the medical field under the perspective of the ISAPP definition: Scientific, regulatory, and marketing considerations. Front. Pharmacol..

[33] Iordache F., Iordache C., Chifiriuc M., Bleotu C., Pavel M., Pelinescu D., Sasarman E., Lazar V., Bucur M., Dracea O. (2008). Antimicrobial and immunomodulatory activity of some probiotic fractions with potential clinical application. Arch. Zootech..

[34] Kimoto-Nira H., Aoki R., Sasaki K., Suzuki C., Mizumachi K. (2012). Oral intake of heat-killed cells of *Lactococcus lactis* strain H61 promotes skin health in women. J. Nutr. Sci..

[35] Lee D.E., Huh C.-S., Ra J., Choi I.-D., Jeong J.-W., Kim S.-H., Ryu J.H., Seo Y.K., Koh J.S., Lee J.-H. (2015). Clinical Evidence of Effects of Lactobacillus plantarum HY7714 on Skin Aging: A Randomized, Double Blind, Placebo-Controlled Study. J. Microbiol. Biotechnol..

[36] Duarte M., Oliveira A.L., Oliveira C., Pintado M., Amaro A., Madureira A.R. (2022). Current postbiotics in the cosmetic market—An update and development opportunities. Appl. Microbiol. Biotechnol..

[37] Vinderola G., Sanders M.E., Salminen S. (2022). The Concept of Postbiotics. Foods.

[38] Puebla-Barragan S., Reid G. (2021). Probiotics in Cosmetic and Personal Care Products: Trends and Challenges. Molecules.

[39] Bermudez-Brito M., Plaza-Díaz J., Muñoz-Quezada S., Gómez-Llorente C., Gil A. (2012). Probiotic mechanisms of action. Ann. Nutr. Metab..

[40] McFarland L.V., Evans C.T., Goldstein E.J.C. (2018). Strain-Specificity and Disease-Specificity of Probiotic Efficacy: A Systematic Review and Meta-Analysis. Front. Med..

[41] Ra J., Lee D.E., Kim S.H., Jeong J.-W., Ku H.K., Kim T.-Y., Choi I.-D., Jeung W., Sim J.-H., Ahn Y.-T. (2014). Effect of oral administration of Lactobacillus plantarum HY7714 on epidermal hydration in ultraviolet B-irradiated hairless mice. J. Microbiol. Biotechnol..

[42] Kim H.M., Lee D.E., Park S.D., Kim Y.-T., Kim Y.J., Jeong J.W., Jang S.S., Ahn Y.-T., Sim J.-H., Huh C.-S. (2014). Oral administration of Lactobacillus plantarum HY7714 protects hairless mouse against ultraviolet B-induced photoaging. J. Microbiol. Biotechnol..

[43] Lee N.Y., Ibrahim O., Khetarpal S., Gaber M., Jamas S., Gryllos I., Dover J.S. (2018). Dermal Microflora Restoration with Ammonia-Oxidizing Bacteria Nitrosomonas Eutropha in the Treatment of Keratosis Pilaris: A Randomized Clinical Trial. J. Drugs Dermatol..

[44] Notay M., Saric-Bosanac S., Vaughn A.R., Dhaliwal S., Trivedi M., Reiter P.N., Rybak I., Li C.C., Weiss L.B., Ambrogio L. (2020). The use of topical *Nitrosomonas eutropha* for cosmetic improvement of facial wrinkles. J. Cosmet. Dermatol..

[45] Onwuliri V., Agbakoba N.R., Anukam K.C. (2021). Topical cream containing live lactobacilli decreases malodor-producing bacteria and downregulates genes encoding PLP-dependent enzymes on the axillary skin microbiome of healthy adult Nigerians. J. Cosmet. Dermatol..

[46] Natsch A., Schmid J., Flachsmann F. (2004). Identification of odoriferous sulfanylalkanols in human axilla secretions and their formation through cleavage of cysteine precursors by a C-S lyase isolated from axilla bacteria. Chem. Biodivers..

[47] Anukam K.C., Macklaim J.M., Gloor G.B., Reid G., Boekhorst J., Renckens B., van Hijum S.A., Siezen R.J. (2013). Genome sequence of Lactobacillus pentosus KCA1: Vaginal isolate from a healthy premenopausal woman. PLoS ONE.

[48] Falholt Elvebakken H., Bruntse A.B., Vedel C., Kjaerulff S. (2023). Topical *Lactiplantibacillus plantarum* LB244R^®^ ointment alleviates skin aging: An exploratory trial. J. Cosmet. Dermatol..

[49] Christensen I.B., Vedel C., Clausen M.-L., Kjærulff S., Agner T., Nielsen D.S. (2021). Targeted Screening of Lactic Acid Bacteria with Antibacterial Activity toward *Staphylococcus aureus* Clonal Complex Type 1 Associated with Atopic Dermatitis. Front. Microbiol..

[50] Elvebakken H.F., Christensen I.B., Vedel C., Kjærulff S. (2024). A proof of concept: Clinical anti-aging efficacy and safety of *Lactiplantibacillus plantarum* LB244R® applied topically in a double-blinded placebo-controlled study. J. Cosmet. Dermatol..

[51] Di Marzio L., Cinque B., De Simone C., Cifone M.G. (1999). Effect of the lactic acid bacterium *Streptococcus thermophilus* on ceramide levels in human keratinocytes in vitro and stratum corneum in vivo. J. Investig. Dermatol..

[52] Di Marzio L., Cinque B., Cupelli F., De Simone C., Cifone M.G., Giuliani M. (2008). Increase of skin-ceramide levels in aged subjects following a short-term topical application of bacterial sphingomyelinase from Streptococcus thermophilus. Int. J. Immunopathol. Pharmacol..

[53] Guéniche A., Bastien P., Ovigne J.M., Kermici M., Courchay G., Chevalier V., Breton L., Castiel-Higounenc I. (2010). *Bifidobacterium longum* lysate, a new ingredient for reactive skin. Exp. Dermatol..

[54] Holz C., Benning J., Schaudt M., Heilmann A., Schultchen J., Goelling D., Lang C. (2017). Novel bioactive from *Lactobacillus brevis* DSM17250 to stimulate the growth of *Staphylococcus epidermidis*: A pilot study. Benef. Microbes.

[55] Tsai W.-H., Chou C.-H., Chiang Y.-J., Lin C.-G., Lee C.-H. (2021). Regulatory effects of *Lactobacillus plantarum*-GMNL6 on human skin health by improving skin microbiome. Int. J. Med. Sci..

[56] Tsai W.-H., Fang Y.-T., Huang T.-Y., Chiang Y.-J., Lin C.-G., Chang W.-W. (2023). Heat-killed *Lacticaseibacillus paracasei* GMNL-653 ameliorates human scalp health by regulating scalp microbiome. BMC Microbiol..

[57] Coderch L., López O., de la Maza A., Parra J.L. (2003). Ceramides and skin function. Am. J. Clin. Dermatol..

[58] Juncan A.M., Moisă D.G., Santini A., Morgovan C., Rus L.-L., Vonica-Țincu A.L., Loghin F. (2021). Advantages of Hyaluronic Acid and Its Combination with Other Bioactive Ingredients in Cosmeceuticals. Molecules.

[59] Lee D.-G., Trujillo M.E., Kang S., Nam J.-J., Kim Y.-J. (2018). *Epidermidibacterium keratini* gen. nov., sp. nov., a member of the family Sporichthyaceae, isolated from keratin epidermis. Int. J. Syst. Evol. Microbiol..

[60] Lee Y.-G., Lee D.-G., Gwag J., Kim M., Kim M., Kim H.-G., Ko J.-H., Yeo H., Kang S., Baek N.-I. (2019). A 1,1′-biuracil from *Epidermidibacterium keratini* EPI-7 shows anti-aging effects on human dermal fibroblasts. Appl. Biol. Chem..

[61] Brosnan M.E., Brosnan J.T. (2007). Orotic acid excretion and arginine metabolism. J. Nutr..

[62] Kim J., Lee Y.I., Mun S., Jeong J., Lee D.-G., Kim M., Jo H., Lee S., Han K., Lee J.H. (2023). Efficacy and Safety of Epidermidibacterium Keratini EPI-7 Derived Postbiotics in Skin Aging: A Prospective Clinical Study. Int. J. Mol. Sci..

[63] Yang S.-J., Lee C.-W., Cha S.Y., Choi J.-W., Lee S. (2021). Skin Barrier Enhancement of Ferment using Lava Seawater and *Lactobacillus plantarum* HDB1234 as a Novel Cosmetic Ingredient. J. Korean Soc. Cosmetol..

[64] Kim K.M., Song J.-W., Lee C.-W., Kim D.-S., Sohn J., Lee S. (2024). Skin Barrier-Enhancing Effects of Dermabiotics HDB with Regulation of Skin Microbiota. J. Microbiol. Biotechnol..

[65] Kim H.-J., Oh H.N., Park T., Kim H., Lee H.G., An S., Sul W.J. (2022). Aged related human skin microbiome and mycobiome in Korean women. Sci. Rep..

[66] Cui H., Feng C., Zhang T., Martínez-Ríos V., Martorell P., Tortajada M., Cheng S., Cheng S., Duan Z. (2023). Effects of a lotion containing probiotic ferment lysate as the main functional ingredient on enhancing skin barrier: A randomized, self-control study. Sci. Rep..

[67] Cui H., Guo C., Wang Q., Feng C., Duan Z. (2022). A pilot study on the efficacy of topical lotion containing anti-acne postbiotic in subjects with mild -to -moderate acne. Front. Med..

[68] Rawlings A.V., Harding C.R. (2004). Moisturization and skin barrier function. Dermatol. Ther..

[69] Lodén M. (2003). Role of topical emollients and moisturizers in the treatment of dry skin barrier disorders. Am. J. Clin. Dermatol..

[70] Huuskonen L., Anglenius H., Ahonen I., Tiihonen K. (2023). Effects of Bacterial Lysates and Metabolites on Collagen Homeostasis in TNF-α-Challenged Human Dermal Fibroblasts. Microorganisms.

[71] Negari I.P., Keshari S., Huang C.-M. (2021). Probiotic Activity of *Staphylococcus epidermidis* Induces Collagen Type I Production through FFaR2/p-ERK Signaling. Int. J. Mol. Sci..

[72] Lee J.-S., Min J.-W., Gye S.-B., Kim Y.-W., Kang H.-C., Choi Y.-S., Seo W.-S., Lee B.-Y. (2024). Suppression of UVB-Induced MMP-1 Expression in Human Skin Fibroblasts Using Lysate of *Lactobacillus iners* Derived from Korean Women’s Skin in Their Twenties. Curr. Issues Mol. Biol..

[73] Lau M., Mineroff Gollogly J., Wang J.Y., Jagdeo J. (2024). Cosmeceuticals for antiaging: A systematic review of safety and efficacy. Arch. Dermatol. Res..

[74] Han J.H., Kim H.S. (2024). Skin Deep: The Potential of Microbiome Cosmetics. J. Microbiol..

[75] De Almeida C.V., Antiga E., Lulli M. (2023). Oral and Topical Probiotics and Postbiotics in Skincare and Dermatological Therapy: A Concise Review. Microorganisms.

[76] Gkitsaki I., Potsaki P., Dimou I., Laskari Z., Koutelidakis A., Giaouris E. (2024). Development of a functional Greek sheep yogurt incorporating a probiotic *Lacticaseibacillus rhamnosus* wild-type strain as adjunct starter culture. Heliyon.

[77] Ashraf R., Shah N.P. (2011). Selective and differential enumerations of *Lactobacillus delbrueckii* subsp. bulgaricus, *Streptococcus thermophilus*, *Lactobacillus acidophilus*, *Lactobacillus casei* and *Bifidobacterium* spp. in yoghurt—A review. Int. J. Food Microbiol..

[78] Baldwin H.E., Bhatia N.D., Friedman A., Eng R.M., Seite S. (2017). The Role of Cutaneous Microbiota Harmony in Maintaining a Functional Skin Barrier. J. Drugs Dermatol..

[79] Maisel A., Waldman A., Furlan K., Weil A., Sacotte K., Lazaroff J.M., Lin K., Aranzazu D., Avram M.M., Bell A. (2018). Self-reported Patient Motivations for Seeking Cosmetic Procedures. JAMA Dermatol..

[80] Draelos Z., Bogdanowicz P., Saurat J.H. (2024). Top weapons in skin aging and actives to target the consequences of skin cell senescence. J. Eur. Acad. Dermatol. Venereol..

[81] Yadav N., Ajmal G., Kumawat D.M., Diwan A. (2023). Probiotic in Cosmetics: A Patents Landscape Study. Int. J. Life Sci. Pharma Res..

[82] Telesetsky A. (2020). UN Food and Agriculture Organization: Exercising Legal Personality to Implement the UN Convention on the Law of the Sea.

[83] Nerini A., Matera C., Stefanile C. (2014). Psychosocial predictors in consideration of cosmetic surgery among women. Aesthetic Plast. Surg..

[84] Ahluwalia J., Fabi S.G. (2019). The psychological and aesthetic impact of age-related hair changes in females. J. Cosmet. Dermatol..

