# Supplementary figures and images for: Cosmeceuticals: A Review of Clinical Studies Claiming to Contain Specific, Well-Characterized Strains of Probiotics or Postbiotics
Source: Nutrients. 2024 Aug 2;16(15):2526. doi: 10.3390/nu16152526 (PMC11314542; doi:10.3390/nu16152526)

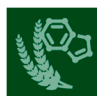

Supplementary Materials  
Flow Chart

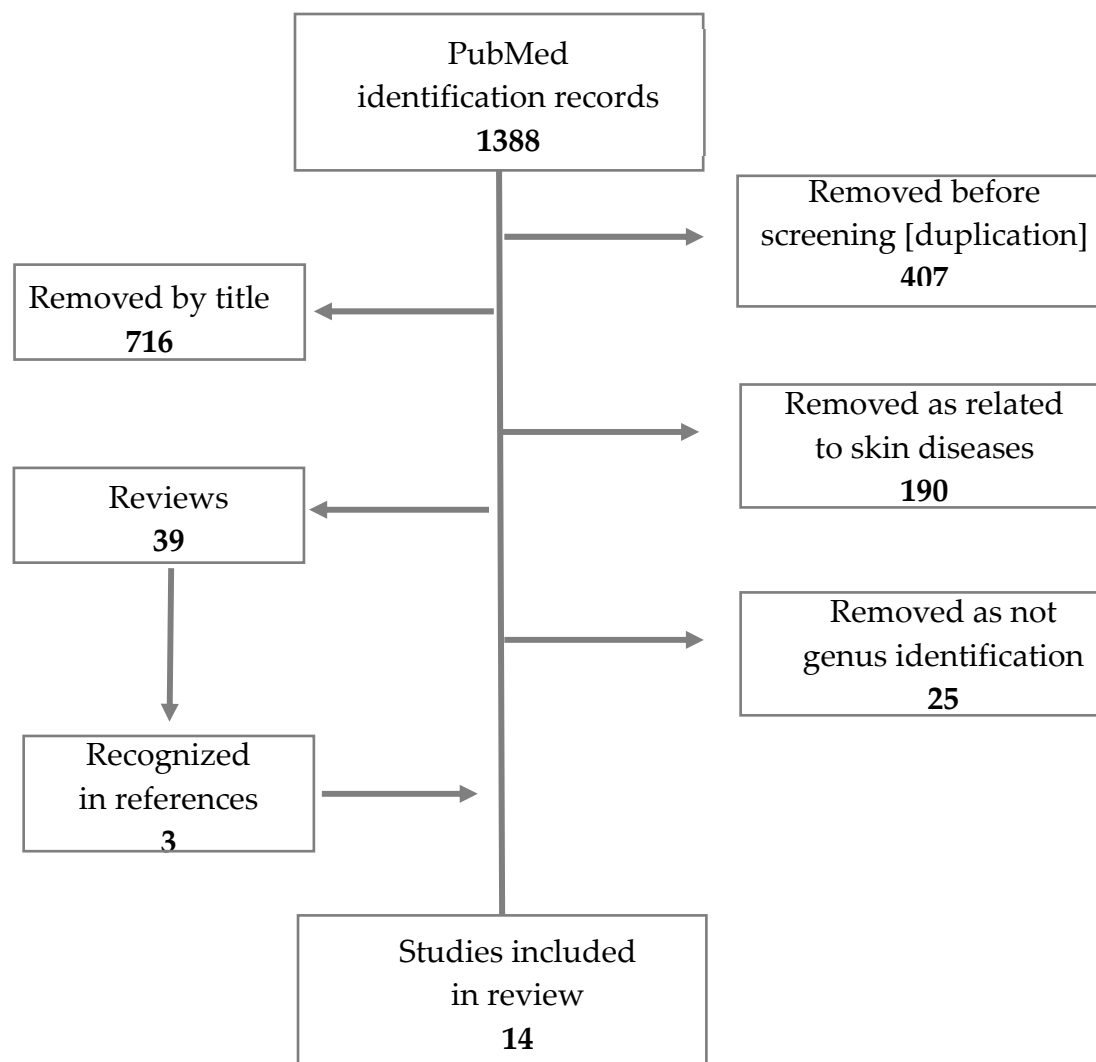

Supplement: Supplementary file 1 [file nutrients-16-02526-s001.zip › nutrients-3099965-supplementary.pdf]
